# The impact of Covid-19 on infertility services and future directions

**DOI:** 10.1530/RAF-20-0017

**Published:** 2020-10-09

**Authors:** I Robertson, A J Kermack, Y Cheong

**Affiliations:** 1 Human Development and Health, University of Southampton, Southampton, UK; 2 Complete Fertility, Princess Anne Hospital, Southampton, UK

## Introduction

The WHO declared COVID-19 a global pandemic on 11 March 2020 and, at the time of writing, SARS-CoV-2 has caused over 23 million infections and 800,000 deaths (Dong *et al.* 2020). In March 2020, consensus recommendations from the European Society of Human Reproduction and Embryology (ESHRE), the American Society of Reproductive Medicine (ASRM) and the British Fertility Society (BFS) advocated a precautionary approach, advising infertility patients planning treatment to avoid becoming pregnant.

At the time, predictive modeling and the surge of hospitalizations in several locations suggested health services could rapidly be overwhelmed and become unable to meet the demand for ventilators and ITU care, resulting in a tsunami of avoidable deaths. The need for urgent action to slow viral transmission was clear; and therefore, infertility services were deemed non-essential and suspended. The UK Human Fertilisation and Embryology Authority (HFEA) directive to this effect was issued on 23 March, the day the UK entered nationwide lockdown. Patients mid-treatment were strongly advised to freeze embryos and those with upcoming cycles were dealt the devastating blow of cancellation; our dynamic and bustling fertility clinics ground to a sudden halt.

## Restarting

Infertility is a serious disease and appropriate treatment is not an optional extra in healthcare (Zegers-Hochschild *et al.* 2017). In extremis, the suspension was necessary, but as the situation improved calls for resumption of services began. Fertility declines irreversibly with age and, in many cases, there is an urgent need to proceed with treatment or risk failure. It is right that the voices of both patients and professionals calling for the restart as soon as safely possible were heard, and resumption of fertility care was prioritised in many countries. The UK government, recognizing the time sensitive and important nature of fertility care, announced on 1 May that fertility centers would be able to apply to restart treatments.

ESHRE, BFS and ARSM have issued detailed guidance on restarting services whilst maximizing safety for patients and staff (ARCS 2020, ASRM 2020, ESHRE 2020). These guidelines include advice on risk-assessment, use of face-coverings, the need to maintain physical distancing and use of personal protective equipment (PPE).

Physical distancing of 1m is associated with a reduction in infection risk of 82% in both healthcare and community settings (adjusted odds ratio (aOR) 0.18, 95% CI 0.09–0.38) (Chu *et al.* 2020). To facilitate distancing, most consultations have moved to video conferencing, with potential to greatly benefit some patients, particularly those for whom regular attendance is inconvenient or difficult. However, this seismic shift is not without problems, as some sensitive consultations are difficult online and virtual consultation skills must be developed.

As we strive to achieve Covid-19 free services and maximize safety, the optimal screening and testing approach remain uncertain, and there is a disagreement between published guidelines (La Marca & Nelson 2020). In the UK, Covid-19 symptom and risk screening questionnaires and rRT-PCR based viral tests for current infection are integral to most protocols, but the inclusion and frequency of Covid-19 testing vary between centers. The BFS recommends rRT-PCR viral test before treatment, if available, and consideration of repeat testing before a surgical procedure, such as oocyte retrieval. Frequent screening and testing of patients and staff could maximize the probability of detecting asymptomatic infection, which may be as high as 42% (Lavezzo *et al.* 2020). However, the effectiveness of this strategy hinges on the tests used being highly sensitive and validated under realistic conditions against a clinically meaningful reference standard (Woloshin *et al.* 2020).

Some centers additionally use antibody tests to detect previous SARS-CoV-2 infection. Despite widespread implementation, the value of antibody testing, other than for population seroepidemiological monitoring, remains unclear. In a Cochrane meta-analysis of studies up until 27/4/2020, the published specificity of SARS-Cov-2 IgG antibody tests were excellent at 99.1% (98.3–99.6), but caution was advised as they had largely been evaluated in patients hospitalized with Covid-19 disease. Extrapolation of accuracy to a younger, infertility patient or staff cohort, who are unlikely to have become severely ill, is less well understood. Sensitivity was highly dependent on time from infection, increasing from 30.1% (95% CI: 21.4 – 40.7) for 1 to 7 days to 96.0% (95% CI 90.6 – 98.3) for 21 to 35 days (Deeks *et al.* 2020). In addition, a recent study demonstrated marked attenuation of antibody detection, particularly in those with asymptomatic proven SARS-CoV-2 infection, 40% of whom were seronegative for IgG antibodies 8 weeks post-infection (Long 2020). Furthermore, the level of protection offered by a positive antibody result, if any, is unclear. Patients and staff with positive antibody results should continue to consider themselves at risk of both infection and transmission and adhere to all relevant infection control policies.

The guidelines all recommend cancellation of treatment if a woman is newly diagnosed with Covid-19 during stimulation, and freeze-all if the diagnosis is suspected or made between oocyte recovery and embryo transfer. As winter approaches, the method of diagnosis of Covid-19 will be crucial, as some people with Covid-19 infection have clear symptoms (such as a continuous cough or high temperature), while others’ symptoms are more subtle and difficult to distinguish from other respiratory illnesses. Clinics will need to make difficult decisions on whether to cancel cycles based solely on symptoms or wait for rRT-PCR results, taking into consideration the possibility of false-negative results and the importance of good sampling technique. Whilst there is ongoing community SARS-CoV-2 transmission a cautious approach for those with possible symptoms is advised. Patients must be aware of guidelines prior to starting cycles, in order to make an informed choice to proceed and to guide their decisions about appropriate precautions to take during treatment.

Many of the existing guidelines are open to interpretation by individual centers. Most clinics probably adopt the guidance that is logistically possible in their clinical set-up, aligns their protocols to their hospital policies and is in accordance with the views of their medical leaders. National and international learned societies and regulatory authorities should continue their concerted efforts in providing consensual guidance. Looking ahead, clinics need to monitor national and local case numbers (GOV.UK) and be prepared for change if there is an escalation of local risk or a need for further lockdowns.

## COVID, fertility and pregnancy

Currently, any potential effects of Covid-19 infection on gametes, embryos, infertility treatments and early gestation remain uncertain. In the SARS-CoV-1 2002–2003 pandemic, miscarriage rates were as high as 57% in the first trimester, although the number of pregnant women affected was very small (Wong *et al.* 2004). Concern regarding SARS-CoV-2 and ART may be justified, as ACE2 and CD147 receptors have been demonstrated on oocytes and pre-implantation embryos (Essahib 2020) and it is these receptors that allow this virus to enter cells. Furthermore, a small study has demonstrated viral RNA present in 6/38 semen samples from men who had recovered from Covid-19 (Li *et al.* 2020). Despite this biological plausibility, there is not currently evidence of sexual transmission or direct infection of either gametes or embryos with SARS-CoV-2 and current guidance recommends routine laboratory practice, with enhanced precautions only for those with the proven disease. It is also unknown whether SARS-CoV-2 causes an increased risk of infertility, fetal anomalies or adverse pregnancy complications, although early data is reassuring. However, data suggest vertical transmission in later pregnancy can occur at low rates, with a review reported at ESHRE 2020 showing 10/688 babies born to infected mothers were rRT-PCR positive, 30% having IgG/IgM antibodies consistent with the transmission in utero (Bahadur 2020). ESHRE, ASRM and the International Federation for Fertility Societies have committed to continuous monitoring of the effect of Covid-19 on gametes and reproductive tissues, collecting data on pregnant patients infected during the pandemic, and assessing the outcomes of mothers and neonates.

UK, French and US surveillance studies have provided some evidence regarding the maternal risk of infection in pregnancy. Pregnant women appear no more likely to contract the infection than the general population (Docherty *et al.* 2020). US data suggests pregnancy was associated with increased risk of hospitalization and ICU admission but not with increased mortality. Hispanic and non-Hispanic black pregnant women appeared to be disproportionately affected by SARS-CoV-2 infection (Ellington 2020). In the UK, of 427 pregnant women admitted to hospital with SARS-CoV-2 infection between 1 March 2020 and 14 April 2020, 402 were in the late second or third trimester and more than half were from BAME groups (Knight *et al.* 2020). An assessment of an individual’s clinical situation and risk profile should be made prior to fertility treatment, considering treatment urgency, medical co-morbidities, age, ethnicity and likely persistence of the virus in the local community in the medium term. As always, avoidance of unnecessary obstetric risk is a duty of reproductive medicine clinicians and, despite the paucity of data of Covid-19 outcomes in multiple pregnancies, there should be careful consideration before transferring more than one embryo. Furthermore, in view of the current uncertainty of the data on safety, alternative options to fertility treatment should be offered and documented, including explicit mention of deferring treatment or freezing all embryos for future use. Clinicians should ensure they are up to date by checking UKOSS (Knight *et al.* 2020) and RCOG (RCOG 2020) publications and future reports from research such as pan-Covid (COVID 2020). A fertility society, such as BFS, providing a succinct summary or webinar of relevant learning for fertility clinicians in response to key publications, would be useful. This approach could minimize variations in the information given to patients.

## Psychological impacts

The psychological burden and emotional upheaval caused by infertility and its treatment outside of the pandemic scenario are known to be immense (Cousineau & Domar 2007). Patients whose tests or treatments were postponed due to Covid-19 were significantly impacted psychologically. In an online questionnaire study of affected patients, 11.9% of respondents reported feeling ‘not able at all’ to cope with the resulting stress (Boivin 2020). To have the chance of achieving parenthood removed, particularly at a time when accessing one’s usual support network was difficult, was very distressing. Many found uncertainty regarding the length of treatment suspension particularly challenging and some vocalized their distress through social and other media avenues. Infertility services tried to ameliorate this pain, with the provision of online counseling, consultations and support. However, services were operating with limited staff and the move to online psychological support made counseling provision harder to deliver and possibly less effective. Open letters to patients were published by the HFEA and fertility service representatives engaged with media outlets (Donnelly & Dixon 2020). As ever, we must reflect on communication with our patients, and evaluate what has worked well and what has not. The intense distress shared online suggests, perhaps, infertility services underestimated the need for regular communication and support.

As we restart services, we must be mindful of the ongoing distress patients may be experiencing and focus on support provision. The exclusion of the partner from many appointments has been advised to allow easier social distancing in clinics. However, partner support throughout the treatment process is a substantial element in relieving the psychological burden. Alternative methods for partner participation, such as the use of phone or video, frequent review of the exclusion policy and relaxation as soon as feasible are indicated.

We should also not underestimate the impact that closing units had on staff. Some were redeployed to assist other clinical services, undertaking mandatory upskilling and retraining; some worked from home, valiantly trying to support distressed and disappointed patients, with few answers on when the hiatus would end; others were furloughed. Following reopening, staff had to deal with their own and patients’ anxiety surrounding contracting Covid-19, patients’ distress at suspended cycles and delays, as well as managing to swiftly alter their practice to incorporate the latest guidance.

## Prioritiation

During treatment suspension, urgent fertility preservation cycles for oncology patients continued. Patients having such cryopreservation cycles may be medically complex, with some requiring anaesthetic support and aerosol-generating procedures for safe oocyte retrieval. Providing this urgent care in the Covid era requires careful risk mitigation for both patient and staff safety, including the provision of appropriate PPE and fit testing for staff. Other patients, for example those older or with low ovarian reserve, face a less acute, but still pressing, need for treatment. Low prognosis patients, as defined by the POSEIDON group, represent approximately 30–50% of patients seeking ART. Some reassurance was provided by a recent retrospective, single-center study comparing a group with diminished ovarian reserve whose IVF cycle started 1–90 days after initial presentation to those starting between 91 and 180 days, which showed no significant difference in live-birth rates (Romanski 2020). In contrast, a large modeling study assessed the potential impact of 1-, 3- and 6-month ART shutdowns on individual prognosis and population live-birth rates, showing a difference, with older women greatest affected by delays in treatment (Smith *et al*. 2020). Prioritization is being encouraged for the extremes but, in practice, these decisions can be medically and ethically complex. When prioritization is required, this should be performed by a multi-disciplinary team with open communication with patients and timescales of delays discussed.

## Impact of economic uncertainty

The global economic impact of Covid-19 is staggering. Currently, the IMF predicts negative 4.9% global growth in 2020, with −10.2% change in UK GDP and has stated ‘Covid-19 has had a more negative impact on activity in the first half of 2020 than anticipated, and the recovery is projected to be more gradual than previously forecast’ (IMF 2020). In a sector with a large private component, widespread economic uncertainty will be damaging, and centers may struggle if there is a reduction in self-funded cycles. In the longer term, it is challenging to predict the many societal effects of Covid-19 and how they will impact infertility care. Household economic uncertainty may delay conception attempts, causing a future spike in demand for infertility services. In the UK, as the government pushes its ‘levelling-up’ agenda, we must continue to highlight the inequalities of current provision for NHS funded fertility treatment. If the government truly recognises the importance of our services, all patients should be treated equally, with eligibility for treatment based on need, not the postcode.

## Lessons learned and future opportunities

This pandemic has starkly revealed both strengths and weaknesses within our society and its systems. Cohesive action has been essential and the professional bodies and societies within infertility care have shown effective leadership, taking timely and decisive actions, even when unpopular. In the initial phases of outbreaks, clinic policies and SOPS were inevitably changing rapidly and new ways of information sharing, such as #medtwitter and webinars, enabled rapid dissemination of experience and learning. We are still at the stage of learning in this pandemic and we must take time to reflect on the safety and efficiency of changes that have been made.

Despite this difficult time, there are positives. Infertility services have demonstrated great resilience, adaptiveness and willingness to transform in order to safely and swiftly restart treatments. Technology has been embraced, enabling consultations to continue via video conferencing and cutting-edge education to reach a worldwide audience, with ESHRE’s 2020 virtual conference and ‘COVID and ART Webinar’ reaching over 14,000 participants combined. As we move beyond this pandemic, the improvements driven by innovation should become the new normal. Expansion of provision for digital learning and collaboration should continue, for example to distil the complexities of Covid-19 research into practically useful information for clinicians.

Finally, throughout society, recognition of the value of medical research is at an all-time high, with a widespread appreciation of the necessity for research into innovative protective equipment, treatments and vaccines to enable an exit from this pandemic and economic recovery. Fertility centers should embrace research and innovation more so than ever, and study design must allow the inclusion of smaller units so that all available data is captured. Participation in Covid-19 related studies is imperative, with the impact of Covid-19 on assisted reproduction outcomes and early pregnancy an important unanswered question. As such, we should aim to maximize enrolment of eligible patients into studies such as PAN-COVID, the US ASPIRE Study, the ESHRE Covid-19 case reporting system and UKOSS. Improved knowledge will bring greater certainty on how best to provide safe and effective infertility services for our patients.

## Declaration of interest

The authors declare that there is no conflict of interest that could be perceived as prejudicing the impartiality of this commentary.

## Author contribution statement

This commentary was planned jointly by all co-authors, Dr Robertson drafted the article, Dr Kermack and Prof Cheong reviewed and provided comments and all have approved the final version for publication.
